# Local Treatment of Hand-Foot Syndrome with Uridine/Thymidine: *In Vitro* Appraisal on a Human Keratinocyte Cell Line HaCaT

**DOI:** 10.1100/2012/421325

**Published:** 2012-07-31

**Authors:** J. Hartinger, P. Veselý, E. Matoušková, S. Argalacsová, L. Petruželka, I. Netíková

**Affiliations:** ^1^Hospital Pharmacy, First Faculty of Medicine, Charles University in Prague and General Teaching Hospital, 12108 Prague, Czech Republic; ^2^Central European Institute of Technology (CEITEC), Brno University of Technology, 60177 Brno, Czech Republic; ^3^Prague Burn Centre, Third Faculty of Medicine, Charles University in Prague, 11000 Prague, Czech Republic; ^4^Oncology Department, Hospital Na Bulovce, 18001 Prague, Czech Republic; ^5^Department of Oncology, First Faculty of Medicine, Charles University in Prague and General University Hospital in Prague, 12108 Prague, Czech Republic; ^6^Oncology and Clinical Pharmacist, Hospital Na Bulovce, 18001 Prague, Czech Republic

## Abstract

5-fluorouracil (5-FU) is one of the most commonly used antineoplastic drugs in the anticancer therapy. The hand-foot (HF) syndrome (palmar-plantar erythrodysesthesia) is an adverse effect frequently related to long-term i.v. administration of 5-FU or its orally applicable prodrug capecitabine. Its severity can even lead to interruption of the otherwise effective anticancer therapy. Tentative practice in some clinics has shown that topical application of 10% uridine ointment is beneficial for calming down the HF syndrome. This study is focused on verifying the alleged protective activity of uridine in the *in vitro* model of cultured human keratinocyte cell line HaCaT. We also tested the protective effects of thymidine alone or uridine-thymidine combination. The cellular viability time progression was measured in order to evaluate the effect of protective agents by three different types of cytopathogenicity tests—NTCA test (non-destructive test of cellular activity), modified MTT test and RTCA (real-time cell analyser, Roche). All three methods proved the ability of uridine and uridine-thymidine combination to protect keratinocytes against 5-FU damage *in vitro*. While thymidine alone did not show any remarkable effect, the thymidine-uridine combination demonstrated enhanced protective activity compared to uridine alone. Our findings provided the supporting rationale for using uridine or uridine-thymidine ointments in the HF syndrome local therapy.

## 1. Introduction

 5-fluorouracil (5-FU) is one of the most frequently used chemotherapeutic agents in the treatment of various tumour diseases such as breast, oesophagus, and colorectal cancer [1, 2]. There is hardly any therapeutic schedule for colorectal cancer not containing 5-FU or its orally administered prodrug capecitabine.

 5-FU continuous infusion shows better tolerability and efficacy than its bolus administration [3]. One of the most remarkable adverse effects accompanying long-term 5-FU administration is the hand-foot syndrome (palmar-plantar erythrodysesthesia), which represents locally misplaced activity of 5-FU systemic therapy [3, 4]. It is also one of the most frequent adverse effects of capecitabine [5], which often replaces i.v. 5-FU in therapeutic schedules. It can be characterized as a nonspecific toxic reaction of keratinocytes to the presence of a cytotoxic agent, not necessarily fluoropyrimidine only. The rate of clinical manifestation can be divided into four grades from slight dysesthesia to desquamation, blistering, and ulceration [6], with possible resulting interruption of an otherwise effective therapy of the main disease [6, 7].

 Empirical clinical practice in Germany, Poland, and Czech Republic showed that topical application of 10% uridine ointment can help prevent the HF syndrome by local cancellation of the 5-FU effect [8–11]. The protective effect of uridine was also reported in the study of mice treated with 5-FU in which uridine infusions significantly reduced the systemic toxicity of 5-FU [12]. This was not confirmed by another study in which uridine after i.v. administration to the patients was rapidly catabolized and did not prevent the side effects of 5-FU therapy [13]. On the other hand, orally administered uridine triacetate, which increased the intracellular concentration of uridine, was successfully used as an antidote in cases of 5-FU overdose [14].

 Another agent modulating 5-FU toxicity is thymidine. It abrogates the inhibition of thymidylate synthase (TS) by 5-FU [15, 16]. In some studies, the combination of 5-FU and thymidine led to the decreased 5-FU toxicity* in vitro*, but in other studies the outcome was opposite [17, 18].

 This study is focused on finding out whether there is also convincing evidence for the alleged protective activity of uridine and thymidine in the *in vitro* model of cultured human skin cells. We also additionally examined the combination of these two agents. For that purpose we used human keratinocyte cell line HaCaT [18] and three different methods for evaluating cell viability *in vitro*. NTCA test is based on evaluating the cell morphology and cell density of surviving cells [19], the modified MTT test shows metabolic activity in time [20], and a rather novel technology of Real-Time Cell Analyzer (RTCA, Roche) continuously evaluates cell viability [21] and was already successfully used for the study of HaCaT cells [22].

## 2. Materials and Methods

### 2.1. Cultivation of HaCaT Keratinocytes

HaCaT cells are defined as a spontaneously immortalized human epithelial cell line that maintains full epidermal differentiation capacity [18]. The cells were cultured in H-MEM medium supplemented with nonessential amino acids, 0.12 g/L sodium pyruvate, 1 g/L NaHCO3, 10% bovine serum, 2% fetal bovine serum, and antibiotics (200 U/mL penicillin and 100 *μ*g/mL streptomycin). The cells were maintained in humidified atmosphere at 37°C and 3.5% CO_2_.

### 2.2. NTCA Test

As the first method for measuring cell viability we used the non-destructive test of cellular activity NTCA [19]. The cells were plated into the 24-well plate (40,000 cells per well in one mL of the medium with phenol red). When cellular layers nearly reached confluence, one ml of the tested agents dissolved in the medium was added. After 5 days, the medium was washed out and the cells were stained by May-Grünwald and Giemsa-Romanowski solutions. The photographs of the whole plates show the presence of the cells in the wells and microscopic morphology shows the degree of damage. The viability was estimated according to the fraction of the cells that remained attached to the culture dish and according to the cell morphology.

### 2.3. RTCA (xCELLigence)

For the second cell survival measurements, we used a Real-Time Cell Analyser produced by Roche Applied Sciences (xCELLigence). Seven thousand cells in 100 *μ*L medium were plated into each of the wells of a 96-well plate. Each well is provided with golden electrodes on the bottom surface. The measurable impedance between these two electrodes grows when the cells are growing and dividing. Cell surface changes, adhesion, and morphology also play a role in this measurement. As a result, we obtain the “cell index” derived from the above-mentioned cellular properties. The cell index can be generally considered as the cellular viability indicator with some limitations [21].

When the cell index plot curves were growing exponentially, the tested substances diluted in 100 *μ*L of the medium were added. We have found that exponential growth of the cell index curve occurs when the cells are slightly subconfluent. The cell index value was recorded every 15 minutes. In the presented plot, time 0 represents the time of adding the tested compounds.

### 2.4. MTT Assay Modified for Measuring Time Progression of Cellular Metabolic Activity

The third method used was the classical endpoint MTT test [20] modified for measuring the time progression of cellular metabolic activity. The cells were plated into 96-well plates (12,000 cells per well in 100 *μ*L of medium without phenol red). The outer wells were not seeded with cells but filled with sterile water for injection. Two plates were used for each experiment. When cellular layers reached confluence, 100 *μ*L of the tested agents dissolved in the medium were added to the wells. One plate was used for two different settings (different tested substances or their combinations). We recorded the absorbance values every day for six days. Each day 10 *μ*L of MTT (5 mg/mL, dissolved in PBS) was added to one column in the plate. After six hours of incubation, the formazan production was stopped by 100 *μ*L of 10% SDS solution in distilled water. After overnight incubation, the plates were analysed by ELISA reader (570 nm test wavelength and 630 nm background wavelength). The mean values of absorbance from the wells with the same concentration of the tested agents (three for each concentration and each day) were considered as indicators of the cellular metabolic activity.

## 3. Results

The NTCA test was performed to find out whether uridine can protect keratinocytes against 5-FU damage and, if so, which concentrations of 5-FU and uridine are meaningful for further testing.

**Figure 1:**
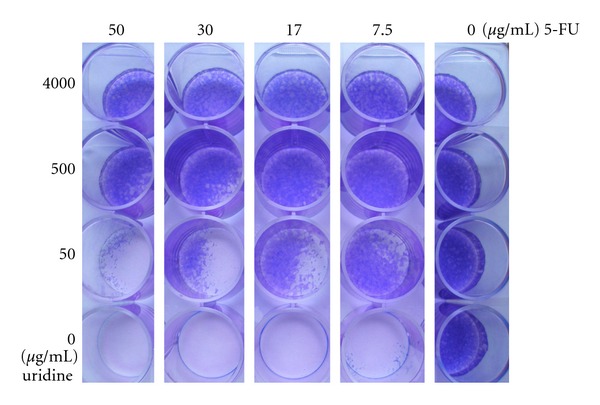
NTCA test. HaCaT cells after 5-day exposition to 5-FU and uridine. 40,000 cells were seeded to each well of the plate. After reaching confluency, 5-FU and uridine were added in different concentrations. After 5 days, the medium was washed out and the cells were stained by May-Grünwald and Giemsa-Romanowski solutions.

The results of NTCA test proved the ability of uridine to protect the cells from damage caused by 5-FU. Therefore, we tested several concentrations of 5-FU and uridine (Figure 1). The lowest concentration (7.5 *μ*g 5-FU/mL) is still higher than steady plasma concentration of any dosage schedule of this drug or its prodrugs used in clinical praxis [23–25]. Wide range of concentrations was chosen for uridine. The cultivation was stopped after 5 days.

Figure 1 shows that all the cells died when no uridine was added in all concentrations of 5-FU applied (7,5–50 *μ*g 5-FU/mL). The differences between uridine concentrations are clearly expressed in the cell survival. In the concentration 50 *μ*g/mL of uridine, the cells showed less viability in wells with higher concentrations of 5-FU. In higher concentrations of uridine, the majority of cells survived no matter how high the 5-FU concentration was.

The results from NTCA test clearly visually demonstrates the uridine protection efficacy for the cells treated with 5-FU but did not provide the information about time progression of this protective effect.

**Figure 2:**
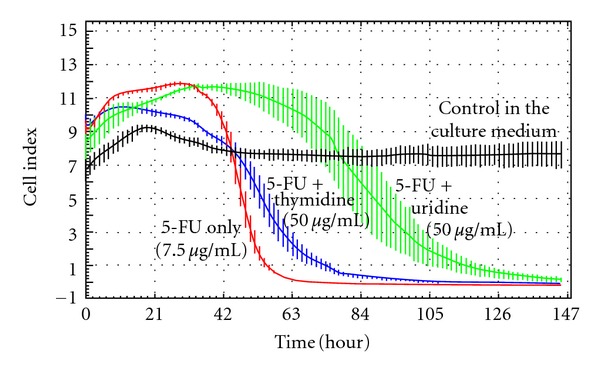
RTCA measurement. Comparison of the protective effect of uridine and thymidine in the presence of 7.5 *μ*g 5-FU/mL. The curves represent the course of HaCaT cell viability after 147 hours of exposure to 5-FU (7.5 *μ*g/mL) only (red curve) or with protective agents: green (5-FU + uridine 50 *μ*g/mL), blue (5-FU thymidine 50 *μ*g/mL). The black curve represents control cells in the culture medium.

**Figure 3:**
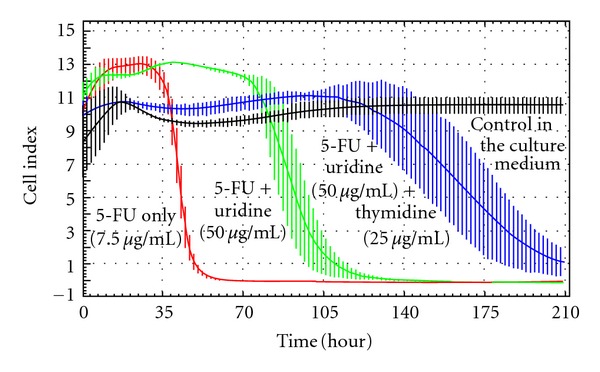
RTCA measurement. Comparison of the protective effect of uridine only and uridine together with thymidine in the presence of 7.5 *μ*g 5-FU/mL. The curves represent the course of HaCaT cell viability after 210 hours of exposure to 5-FU (7.5 *μ*g/mL) only (red curve) or with protective agents: green (5-FU + uridine 50 *μ*g/mL), blue (5-FU + uridine 50 *μ*g/mL and thymidine 25 *μ*g/mL). The black curve represents control cells in the culture medium.

**Figure 4:**
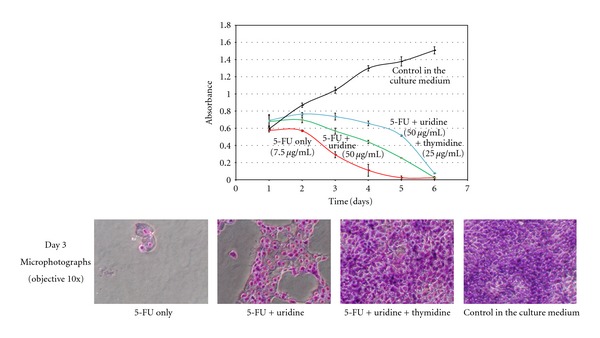
MTT test. Comparison of the protective effect of uridine only and uridine together with thymidine in the presence of 7.5 *μ*g 5-FU/mL. The curves represent the course of HaCaT cell viability after 6 days of exposure to 5-FU (7.5 *μ*g/mL) only (red curve) or with protective agents: green (5-FU + uridine 50 *μ*g/mL), blue (5-FU + uridine 50 *μ*g/mL and thymidine 25 *μ*g/mL). The black curve represents control cells in the culture medium. Microphotographs of HaCaT cells stained with May-Grünwald/Giemsa-Romanowski (day 3, objective 10x) demonstrate the resultant state of the outgrowth of the cell population.

For further confirmation of uridine protective efficacy and monitoring its time progression we used RTCA measurements (Figures 2 and 3) and modified MTT test (Figure 4). According to results from NTCA we used 50 *μ*g/mL of uridine and 7.5 *μ*g/mL of 5-FU. Furthermore, we tested also thymidine to compare its protective effect with uridine.

Figure 2 shows the comparison of protective effect of uridine and thymidine in the concentration 50 *μ*g/mL. Thymidine showed much lower protective ability.

Another RTCA measurement shows that combination of uridine and thymidine together in the ratio 2 : 1 (50 *μ*g of uridine/mL and 25 *μ*g of thymidine/mL) protects the cells better than uridine only. This ratio was chosen according to our findings from former testing of several ratios of these two agents in three different 5-FU concentrations (results not presented).

The results of RTCA test presented in Figure 3 show clearly that the cells with uridine protection survived 60 hours longer than the cells without any protective agent. The cells with uridine-thymidine combination survived 120 hours longer than the cells without any protective agent.

The results of MTT test presented in Figure 4 confirm that uridine is able to protect the cells against 5-FU damage and that the combination of uridine and thymidine protects the cells better than uridine only until day 5.

## 4. Discussion

The hand-foot syndrome often represents a serious complication of fluoropyrimidine-based chemotherapy, accompanying especially its long-term administration and anticancer treatment. There are no generally accepted recommendations for effective treatment of the HF syndrome. One of the approaches, already clinically tested on a small scale, is topical application of 10% uridine ointment [8, 9, 11].

The 5-FU concentrations in the skin of the palms and soles of patients with 5-FU-induced HF syndrome are difficult to predict. The fact that this syndrome occurs almost exclusively with 5-FU continual administration suggests that some concentration mechanism may be involved. On the other hand, Fischel and Formento showed that 5-FU and its prodrugs and metabolites are more toxic to the HaCaT keratinocytes than to colorectal cancer cells [26].

In RTCA and MTT experiments we used the 5-FU concentration of 7.5 *μ*g/mL. This concentration is higher than the steady-state plasma concentration of any dosage schedule of 5-FU or its prodrugs used in clinical practice, which ranges from 9 ng/mL to 950 ng/mL [23–25]. We proved the ability of uridine to protect *in vitro *cultured keratinocytes against cytotoxic damage caused by 5-FU exposure. The results were proved by three different *in vitro* methods (NTCA, RTCA, and MTT). The phase contrast microscope also showed differences in the cell damage.

This brings up the question of potential systemic effect of uridine absorbed from the ointment. The partition coefficient Log P_octanol/water_  for uridine is −1.98 whilst partition coefficients between 1 and 3 refer to molecules that are suitable for systemic absorption through the skin [27]. It is therefore unlikely that uridine could be systemically absorbed from the ointment in a significant amount. Furthermore, the i.v. administered uridine has a distribution volume approximately similar to whole body water and is catabolized rapidly with 2-hour half-life [13]. Therefore, we do not expect that absorption of a small amount of uridine from the locally administered 10% ointment should affect the antineoplastic therapy.

We also tested the combination of uridine and thymidine in the ratio 2 : 1. This combination results in markedly better protection than uridine alone. The rationale for this may be that uridine competes with 5-flurouridine for RNA incorporation and thymidine does the same in the case of DNA. Moreover, the inhibition of TS is abrogated by thymidine substitution, and therefore the effect of this nucleoside deficiency leading to an inappropriate DNA synthesis is prevented [15].

## 5. Conclusion

We succeeded in prolonging the time of onset of cell death by 5-6 days using uridine or uridine-thymidine treatment in the presence of 7.5 *μ*g/mL of 5-FU. This is in accordance with the effect observed in empirical clinical practice using the application of uridine ointment [8, 9, 11]. The presented results provide preclinical confirmation of the uridine ointment usage meaningfulness and rationale for further large-scale clinical testing. These results also show that the HaCaT keratinocyte model is suitable for measuring the effect of protective agents on skin integrity at least in the case of 5-FU-induced HF syndrome.
